# Immunohistochemical detection improves the prognostic value of lymphatic and blood vessel invasion in primary ductal breast cancer

**DOI:** 10.1186/1471-2407-14-676

**Published:** 2014-09-18

**Authors:** Fadia J A Gujam, James J Going, Zahra M A Mohammed, Clare Orange, Joanne Edwards, Donald C McMillan

**Affiliations:** Academic Unit of Surgery, College of Medical, Veterinary and Life Sciences-University of Glasgow, Royal Infirmary, Glasgow, UK; University Section of Pathology, College of Medical, Veterinary and Life Sciences-University of Glasgow, Southern General Hospital, Glasgow, UK; University Departments of Pathology, Faculty of Veterinary Medicine, Omar, Almukhtar University, Al bayda, Libya; Wolfson Wohl Cancer Research Centre, Institute of Cancer Sciences, College of Medical, Veterinary and Life Sciences-University of Glasgow, Glasgow, UK

## Abstract

**Background:**

Lymphovascular invasion (LBVI) including lymphatic (LVI) and blood (BVI) vessel invasion is a critical step in cancer metastasis. In breast cancer, the optimal detection method of LBVI remains unclear. This research aimed to compare the prognostic value of different assessments of the LVI and BVI in patients with early breast cancer.

**Methods:**

The study cohort included 360 patients with a median follow-up of 168 months. LBVI on H&E sections (LBVI_H_&_E_) was reviewed centrally and blinded to the pathology report. Immunohistochemical staining for D2-40 and Factor VIII was performed to identify LVI_D2–40_ and BVI_FVIII_.

**Results:**

LBVI_H_&_E_, LVI_D2–40_ and BVI_FVIII_ were present in 102 (28%), 127 (35%) and 59 (16%) patients respectively. In node-negative patients (206), LBVI_H_&_E_, LVI_D2–40_ and BVI_FVIII_ were present in 41 (20%), 53 (26%) and 21 (10%) respectively. In triple-negative patients (120), LBVI_H_&_E_, LVI_D2–40_ and BVI_FVIII_ were present in 35 (29%), 46 (38%) and 16 (13%) respectively. LBVI_H_&_E_ was significantly associated with tumour recurrence in the whole cohort (*P* < 0.001), node-negative patients (*P* = 0.001) and triple-negative patients (*P* = 0.004). LVI_D2–40_ and BVI_FVIII_ were significantly associated with tumour recurrence in whole cohort, node-negative (all *P* < 0.001) and triple-negative patients (*P* = 0.002). In multivariate survival analysis, only LVI_D2–40_ and BVI_FVIII_ were independent predictors of cancer specific survival in the whole cohort (*P* = 0.023 and *P* < 0.001 respectively), node-negative patients (*P* = 0.004 and *P* = 0.001 respectively) and triple-negative patients (*P* = 0.014 and *P* = 0.001 respectively).

**Conclusion:**

Assessment of LVI and BVI by IHC using D2-40 and Factor VIII improves prediction of outcome in patients with node-negative and triple-negative breast cancer.

## Background

Breast cancer is a common cancer in female and one of the leading causes of cancer death in women. It accounts for approximately one tenth of all new cancers and a quarter of all female cancer cases [1]. In the UK more than 49,000 women diagnosed with breast cancer in 2011 accounting for 30% of female cancer incidence. However, the survival rate has improved with 78% surviving 10 or more years [2].

Lymphovascular invasion (LBVI) including lymphatic (LVI) and blood (BVI) vessel invasion is a critical step in cancer metastasis. It refers to the invasion of tumour cells into endothelium-lined lymphatic and/or blood vessels [3, 4]. In breast cancer LBVI has been recognised more than four decades ago [5]. Since then, a number of independent studies have investigated the prognostic value of LBVI in node-negative and node-positive breast cancer [6–17].

The College of American Pathologists (CAP) consensus (1999) and 11th St Gallen meeting (2009) did not agree on the need for specific stains to identify vascular spaces or to distinguish specifically between LVI and BVI [18, 19]. Although staging guidelines of the American Joint Cancer Committee on Cancer (2005) mandates distinguishing between lymphatic and blood vessel invasion, these guidelines lack a routine standardised and objective assessment method to reliably differentiate them [20]. It remains a challenge to distinguish true LVI and BVI from retraction artifacts caused by tissue handling, fixation and processing on haematoxylin and eosin (H&E) stained sections [21–23].

Numerous studies have reported that LBVI and LVI are powerful prognostic factors of poorer survival in patients with early breast cancer using both H&E and IHC approaches [24]. While immunohistochemistry (IHC) appears more reliably to detect LBVI and LVI than H&E, the prognostic role of BVI and optimal detection methods remain unclear [24].

The aim of the present study was to examine the prognostic value of different assessments of LVI and BVI in patients with early, and in particular node-negative and triple-negative breast cancers.

## Methods

### Patients

Patients with invasive ductal breast cancer, who had undergone surgery between the years 1995 to 1998 at Royal Infirmary, Western Infirmary, Victoria or Stobhill Hospitals, Glasgow, and had formalin-fixed paraffin-embedded blocks of the primary tumour available for evaluation were studied (*n* = 360). Clinicopathological data including age, histological tumour type, grade, tumour size, lymph node status, adjuvant treatment (hormonal therapy and chemotherapy) were retrieved from the routine reports. ER and PR status, using tissue microarrays, were assessed according to the American Society of Clinical Oncology and College of American Pathologists guidelines with cut-off value of 1% positive tumour nuclei [25]. HER2 status were assessed visually using tissue microarrays as previously described i.e. a score 3+ is regarded as positive; 2+ is regarded as equivocal, leading to referral for HER2 FISH; and 0 and 1+ are regarded as negative [26].

The patients included in this study did not receive neoadjuvant therapy or adjuvant anti-HER-2 therapy. The inclusion of ductal breast cancers only was to limit the potential confounding effects of other tumour types on the analysis in the present study.

Patients were routinely followed up following surgery. Date and cause of death was crosschecked with the cancer registration system and the Registrar General (Scotland). Death records were complete until 31st of May 2013 and that served as the censor date. Cancer recurrence was measured from the date of primary surgery until the date of first recurrence of breast cancer. Cancer specific survival was measured from the date of primary surgery until the date of death from breast cancer.

The Research Ethics Committee of North Glasgow University Hospitals approved the use of human tissue in this study.

### Immunohistochemistry

For visualization of lymphatic and blood vessels, 2 consecutive samples of 2.5 μm thick sections from each block (one block/case) were stained for the lymphatic endothelial marker D2-40 (Covance, Monoclonal Antibody, SIG-3730, USA) diluted 1:100 and Factor VIII (Mouse Monoclonal Antibody, NCL-L-Vwf, Leica, Newcastle, UK) diluted 1:100. Sections were dewaxed in xylene and rehydrated through descending concentrations of ethanol. For antigen retrieval of Factor VIII, sections were microwaved for 14 minutes in sodium citrate buffer (pH 6). Endogenous hydrogen peroxidase activity was blocked with 3% H2O2 for 15 minutes. Non-specific binding was blocked by incubation with 10% horse serum for 30 minutes. Sections were subsequently incubated with the respective primary antibody; 60 minutes at room temperature for D2-40 and 30 minutes at 25°C for Factor VIII. Sites of binding were detected using the Envision technique (Dako, code K5007) with 3–30 diaminobenzidine (Vector, code SK 4001, Burlingame, CA, USA), as chromogenic substrate, according to the manufacturer’s instruction. Slides were counterstained with haematoxylin were dehydrated and mounted with DPX. Two full sections of tonsil tissue were used as positive and negative controls for each antibody.

### Slide scanning and scoring

Stained sections with H&E, D2-40 and Factor VIII were scanned at objective magnification × 20 by Hamamatsu NanoZoomer (Hertfordshire, UK). Assessment of LBVI_H_&_E,_ LVI_D2-40_ and BVIFactorVIII were carried out on a computer monitor using the Slidepath Tissue IA system version 3.0 (Slidepath, Leica Biosystems).

### Assessment of LBVI, LVI and BVI

LBVI on H&E sections (LBVI_H_&_E_) was reviewed centrally and blinded to the pathology report. For the assessment of LVI_D2–40_ and BVI_FVIII_ serial sections, similar to that of H&E sections, from each block were stained with D2-40 and Factor VIII. LBVI_H_&_E_, LVI_D2–40_ and BVI_FVIII_ were identified at peritumoural, invasive front or intratumoural areas. LBVI_H_&_E_ was identified using criteria previously described [6], as the presence of tumour cell emboli within a vessel space, which were identified by associated fibrin clot and/or an endothelial cell lining. LVI_D2–40_ was identified by tumour cells within D2-40-positively stained vessels, while BVI_FVIII_ was counted only when tumour cells were identified in D2-40-negative, Factor VIII-positive vessels. A total of 30% of H&E and IHC stained sections for LBVI, LVI and BVI were independently scored by two observers (FJAG, ZMAM) blinded to patient outcome and the other observer’s score. The inter class correlation coefficient (ICCC) of ≥0.84 was obtained for H&E, D2-40 and Factor VIII indicated excellent agreement, and FG scored all the slides and this data was used in the analysis.

### Statistical analysis

Consistency between the observers was analysed using the ICCC. Interrelationships between variables were assessed using contingency table analysis with *X*^*2*^ test for trend as appropriate. Univariate and multivariate survival analysis were performed using the Kaplan-Meier analysis and Cox proportional hazards model with a stepwise backward elimination to derive a final model of variables with a significant independent relationship with survival. All statistical analyses were 2-sided with significance defined as a *P* value <0.05. Deaths up to May 2013 were included in the analysis. All statistical analysis was performed using the SPSS software version 19 (SPSS Inc., Chicago, IL, USA).

## Results

### Clinico-pathological characteristics and LBVI_H_&_E_, LVI_D2-40_ and BVI_FVIII_ in the whole cohort, in node-negative patients and in triple-negative patients

The clinical and pathological characteristics of the 360 patients are shown in Table 1. Majority of patients were older than 50 years (65%), had tumours size less than 2 cm (51%), had grade III carcinoma (52%) and no axillary lymph node involvement (57%). A total of 189 patients (53%) had ER positive tumours and 166 patients (46%) had PR positive tumours. Two hundred eighty nine patients (80%) had HER2 negative tumours with 33% of patients had triple-negative tumours. 81 patients received endocrine based treatment (22%) and 144 received chemotherapy (40%). No information on chemotherapy was available on 7 patients (2%). Eighty nine patients (24%) experienced recurrences. Of these patients, 17 (5%) had local recurrence, 67 (19%) had distant recurrence and 5 patients had both.

**Table 1:** **The clinico-pathological characteristics of patients with primary operable invasive ductal breast cancer (n = 360)**

Clinico-pathological characteristics	Patients, n (%)
Age (≤50/ >50 years)	125(35%)/235(65%)
Size (≤20/ 21-50/ >50 mm)	185(51%)/162(45%)/13(4%)
Grade (I / II / III)	48(13%)/124(34%)/188(52%)
Involved lymph node (-ve/+ve)	206(57%)/154(43%)
ER status (no/yes)	171(47%)/189(53%)
PR status (no/yes)	194(54%)/166(46%)
HER2 status (no/yes)	289(80%)/71(20%)
Triple-negative tumours (no/yes)	240(67%)/120(33%)
Endocrine therapy (no/yes/unknown)	272(76%)/81(22%)/7(2%)
Chemotherapy (no/yes/unknown)	209(58%)/144(40%)/7(2)
Tumour recurrence (no/local/distant/both)	271(75%)/17(5%)/67(19%)/5(1%)
Alive/cancer death/non cancer death	189(53%)/97(27%)/74(21%)

**Figure 1:**
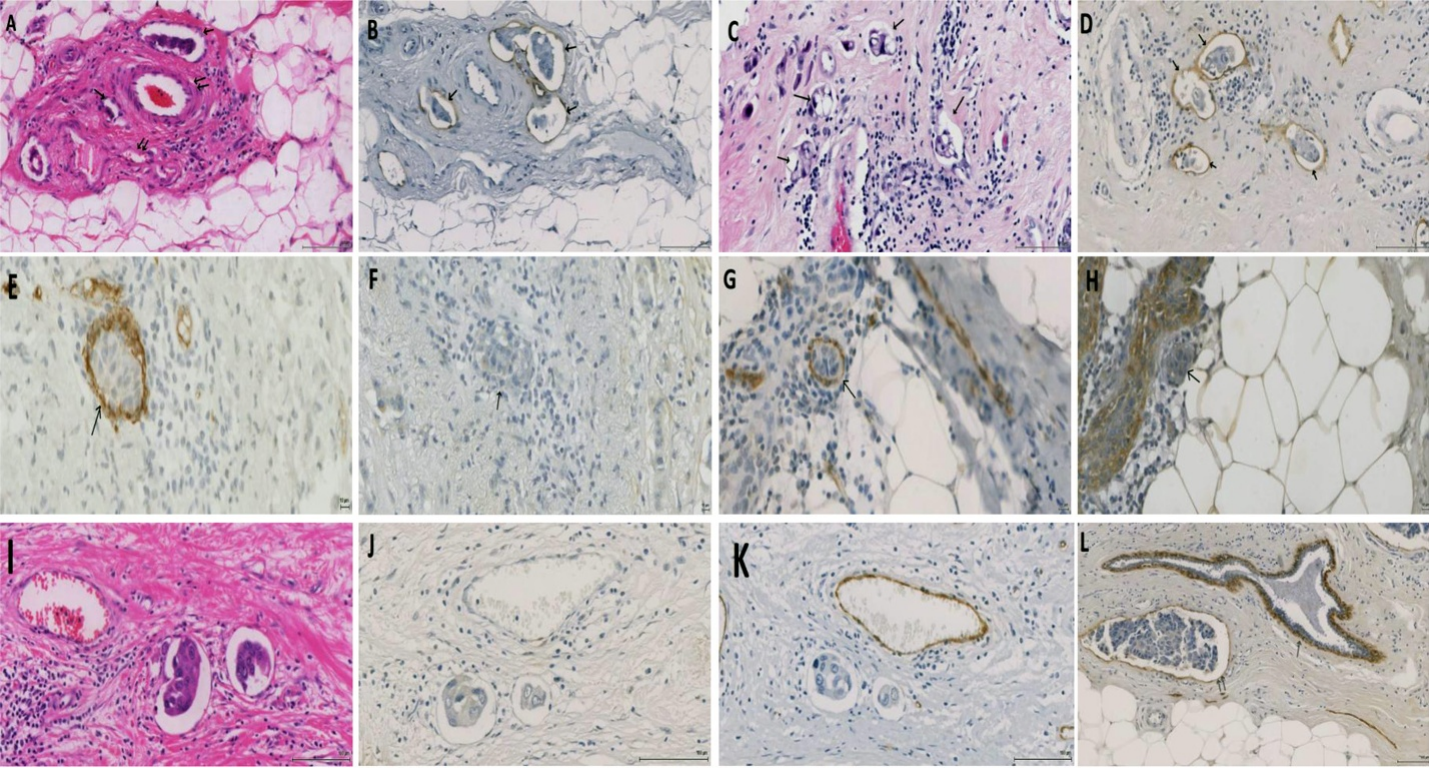
**Examples of LVI and BVI in invasive breast cancer sections stained with H&E, D2-40 and Factor VIII. A**: H&E conspicuous carcinoma emboli in large and small vascular spaces (single arrows) accompanying structurally identified blood vessels (double arrows). **B**: similar section stained with D2-40 confirming that these are LVI (arrows). **C**: carcinoma emboli in small vessels (arrows) that could not be characterised on H&E section. **D**: similar section stained with D2-40 confirming that these are LVI (arrows). (Scale bar 100 μm). **E & G**: carcinoma cells within Factor VIII-positive vessels. These are negative for D2-40 **(F & H)**, indicating BVI. (Scale bar 10 μm). **I-K** show consecutive sections stained with H&E **(I)** showing tumour cells inside endothelial lining space, however, D2-40 **(J)** and Factor VIII **(K)** are both negative suggesting stromal artifact (note positive staining of blood vessel with Factor VIII). **L**: pattern of D2-40 staining in normal breast duct myoepithelium (single arrows) and how it is different from that of lymphatic endothelium (double arrows). (Scale bar100 μm).

LBVI_H_&_E_ was readily identified when tumour cells invaded into large vessels and especially when lymphatic vessels were accompanied by adjacent blood vessels, however, invasion into small lymphatic or blood vessels as well as stromal artifact could be difficult to assess (Figure 1). D2-40 stained vessels were usually clear and readily assessed. LVI_D2–40_ was identified by the presence of tumour emboli in vessels that showed D2-40 positivity of the endothelium. Although D2-40 was positive in myoepithelial cells of breast ducts in some cases, this was readily distinguished from lymphatic endothelium by morphological characteristics (Figure 1L).

D2-40 staining was helpful in identifying small lymphatic emboli and lymphatic vessels obscured by tumour cells (Figure 1). Blood vessels were intensely and continuously positive for Factor VIII. Factor VIII staining of lymphatic endothelium was faint or negative (Figure 1). LVI_D2–40_ was generally more extensive than BVI_FVIII_ and lymphatic tumour emboli were larger than blood vessel emboli.

LBVI_H_&_E_ was reported in 102/360 (28%) patients, LVI_D2-40_ was present in 127/360 (35%) patients and BVI_FVIII_ was present in 59/360 (16%) patients. Eighty nine (25%) patients had LVI only, whereas twenty one (6%) patients had BVI only, and thirty eight (10%) had both LVI and BVI. LBVI_IHC_ (LVI_D2-40_ + BVI_FVIII_) was present in148 (41%) patients. In node-negative patients (206), LBVI_H_&_E_ was present in 41 (20%), LVI_D2-40_ was present in 53 (26%) and BVI_FVIII_ was present in 21 (10%). In triple-negative patients (120), LBVI_H_&_E_ was present in 35 (29%), LVI_D2-40_ was present in 46 (38%) and BVI_FVIII_ was present in 16(13%).

While LBVI_H_&_E_ was strongly associated with LBVI_IHC_ (*P* < 0.001), 80 (22%) patients in whom LBVI_H_&_E_ had not been identified were positive for LVI_D2-40_ and/or BVI_FVIII_. Also, in 34 patients (9%) in whom LBVI_H_&_E_ had been identified, IHC was negative for both LVI_D2-40_ and BVI_FVIII_.

As shown in Table 2, the presence of LBVI_H_&_E_ was associated with large tumour size (*P* < 0.001), high tumour grade (*P* = 0.028), involved lymph node (*P* < 0.001), and tumour recurrence (*P* < 0.001). No association was seen with hormonal status, HER2 status and endocrine therapy however, there was a trend toward increased chemotherapy (0.067). In node-negative patients, only tumour size (*P* = 0.008) and tumour recurrence (*P* = 0.001) were significantly associated with LBVI_H_&_E_. In triple-negative patients, the presence of LBVI_H_&_E_ was associated with tumour size (*P* = 0.025), involved lymph node (*P* =0.009), and tumour recurrence (*P =* 0.004).

**Table 2:** **The inter-relationship between clinico-pathological characteristics and lymphovascular invasion (LBVI**_**H**_&_**E**_ **) in patients with primary operable invasive ductal breast cancer**

All patients (n = 360)	LBVI _H_& _E_ -ve	LBVI _H_& _E_ + ve	( ***P***-value)
	n = 258(72%)	n = 102(28%)	
Age (≤50/ >50 years)	86/175	39/63	0.379
Size (≤20/ 21-50/ >50 mm)	147/106/5	38/56/8	<0.001
Grade (I / II / III)	38/95/125	10/29/63	0.028
Involved lymph node (-ve/+ve)	165/93	41/61	<0.001
ER status (no/yes)	119/139	52/50	0.406
PR status (no/yes)	137/121	57/45	0.634
HER2 status (no/yes)	211/47	78/24	0.254
Tumour recurrence (no/local/distant/both)	213/7/36/2	58/10/31/3	<0.001
Endocrine therapy (no/yes)	191/63	81/18	0.184
Chemotherapy (no/yes)	158/96	51/48	0.067
Alive/cancer death/non cancer death	148/54/56	41/43/18	0.158
Cancer specific survival (months)^a^	178(171–188)	138(121–155)	<0.001
**Node-negative patients (n = 206)**	**n = 165(80%)**	**n = 41(20%)**	
Age (≤50/ >50 years)	51/114	16/25	0.322
Size (≤20/ 21-50/ >50 mm)	103/60/2	17/22/2	0.008
Grade (I / II / III)	29/60/76	5/13/23	0.233
ER status (no/yes)	40/53	32/29	0.252
PR status (no/yes)	47/46	34/27	0.529
HER2 status (no/yes)	138/27	30/11	0.123
Endocrine therapy (no/yes)	118/46	31/9	0.479
Chemotherapy (no/yes)	113/51	29/11	0.658
Tumour recurrence (no/local/distant/both)	143/6/15/1	27/2/10/2	0.001
Alive/cancer death/non cancer death	104/23/38	20/12/79	0.365
Cancer specific survival (months)^a^	190(181–199)	168(146–190)	0.010
**Triple-negative patients (n = 120)**	**n = 85(71%)**	**n = 35(29%)**	
Age (≤50/ >50 years)	31/54	19/16	0.073
Size (≤20/ 21-50/ >50 mm)	49/34/2	14/17/6	0.025
Grade (I / II / III)	1/14/70	0/7/28	0.888
Involved lymph node (-ve/+ve)	56/29	14/21	0.009
Tumour recurrence (no/local/distant)	69/1/15	19/2/14	0.004
Endocrine therapy (no/yes)	76/8	31/4	0.754
Chemotherapy (no/yes)	36/48	15/20	0.999
Alive/cancer death/non cancer death	50/20/15	16/17/2	0.936
Cancer specific survival (months)^a^	171(155–187)	123(96–150)	0.011

^a^ = Mean (95% CI).

Table 3 shows that the presence of LVI_D2-40_ was associated with younger age (*P =* 0.006), large tumour size (*P* = 0.038), high tumour grade (*P* < 0.001), involved lymph node (*P* < 0.001), reduced endocrine therapy (*P =* 0.014), increased chemotherapy (*P* = 0.002) and tumour recurrence (*P* < 0.001). In node-negative patients, the presence of LVI_D2-40_ was associated with younger age (*P* = 0.008) large tumour size (*P* = 0.019) and high tumour grade (*P* = 0.002), HER2 negativity (*P* = 0.032) and tumour recurrence (*P* < 0.001). There was borderline association with reduced endocrine therapy (*P =* 0.070), and increased chemotherapy (*P* = 0.070). In triple-negative patients, the presence of LVI_D2-40_ was associated with involved lymph node (*P* = 0.001) and tumour recurrence (*P* < 0.001).

**Table 3:** **The inter-relationship between clinico-pathological characteristics and lymphatic invasion (LVI**_**D2-40**_ **) in patients with primary operable invasive ductal breast cancer**

All patients (n = 360)	LVI _D2–40-ve_	LVI _D2–40+ve_	( ***P***-value)
	n = 233 (65%)	n = 127 (35%)	
Age (≤50/ >50 years)	69/164	56/71	0.006
Size (≤20/ 21-50/ >50 mm)	129/97/7	56/65/6	0.038
Grade (I / II / III)	156/73/13	56/53/21	<0.001
Involved lymph node (0/1-3/ >3)	153/80	53/74	<0.001
ER status (no/yes)	102/131	69/58	0.056
PR status (no/yes)	118/115	76/51	0.095
HER2 status (no/yes)	193/40	96/31	0.099
Endocrine therapy (no/yes)	168/62	104/19	0.014
Chemotherapy (no/yes)	150/80	59/64	0.002
Tumour recurrence (no/local/distant/both)	199/5/28/1	72/12/39/4	<0.001
Alive/cancer death/non cancer death	141/39/53	48/58/21	0.059
Cancer specific survival (months)^a^	186(177–194)	134(120–149)	<0.001
**Node-negative disease (n = 206)**	**n = 153 (74%)**	**n = 53 (26%)**	
Age (≤50/ >50 years)	42/111	25/28	0.008
Size (≤20/ 21-50/ >50 mm)	96/55/2	24/27/2	0.019
Grade (I / II / III)	33/53/67	1/20/32	0.002
ER status (no/yes)	32/48	40/34	0.082
PR status (no/yes)	38/42	43/31	0.189
HER2 status (no/yes)	130/23	38/15	0.032
Endocrine therapy (no/yes)	106/46	43/9	0.070
Chemotherapy (no/yes)	111/41	31/21	0.070
Tumour recurrence (no/local/distant/both)	137/4/11/1	33/4/14/2	<0.001
Alive/cancer death/non cancer death	99/18/36	25/17/11	0.266
Cancer specific survival (months)^a^	198(190–206)	153(131–174)	0.001
**Triple-negative patients (n = 120)**	**n = 74(62%)**	**46(38%)**	
Age (≤50/ >50 years)	26/48	24/22	0.064
Size (≤20/ 21-50/ >50 mm)	42/28/4	21/23/2	0.367
Grade (I / II / III)	1/13/60	0/8/38	0.712
Involved lymph node (-ve/+ve)	52/22	18/28	0.001
Endocrine therapy (no/yes)	65/9	42/3	0.336
Chemotherapy (no/yes)	36/38	15/30	0.103
Tumour recurrence (no/local/distant)	64/0/10	24/3/19	<0.001
Alive/cancer death/non cancer death	48/13/13	18/24/4	0.217
Cancer specific survival (months)^a^	176(161–192)	122(96–147)	<0.001

^a^ = Mean (95% CI).

Table 4 shows that the presence of BVI_FVIII_ was associated with large tumour size (*P* < 0.001), high tumour grade (*P* = 0.044), involved lymph node (*P* < 0.001), HER2 negativity (*P* = 0.003) and tumour recurrence (*P* < 0.001). There was no association with hormonal status or treatment received. In node-negative patients, BVI_FVIII_ was only significantly associated with larger tumour size (*P* = 0.002) and tumour recurrence (*P* < 0.001). In triple-negative patients, the presence of BVI_FVIII_ was significantly associated with tumour size (*P* = 0.037), involved lymph node (*P* = 0.019) and tumour recurrence (*P* = 0.002).

**Table 4:** **The inter-relationship between clinico-pathological characteristics and blood vessel invasion (BVI**_**FVIII**_ **) in patients with primary operable invasive ductal breast cancer**

All patients (n = 360)	BVI _FVIII_ -ve	BVI _FVIII_ + ve	( ***P***-value)
	n = 301(84%)	n = 59(16%)	
Age (≤50/ >50 years)	104/197	21/38	0.848
Size (≤20/ 21-50/ >50 mm)	168/123/10	17/39/3	<0.001
Grade (I / II / III)	45/104/152	3/20/36	0.044
Involved lymph node (-ve/+ve)	185/116	21/38	<0.001
ER status (no/yes)	139/162	32/27	0.258
PR status (no/yes)	158/143	36/23	0.230
HER2 status (no/yes)	250/51	39/20	0.003
Endocrine therapy (no/yes)	226/70	46/11	0.475
Chemotherapy (no/yes)	178/118	31/26	0.419
Tumour recurrence (no/local/distant/both)	243/13/42/3	28/4/25/2	<0.001
Alive/cancer death/non cancer death	179/60/62	10/37/12	<0.001
Cancer specific survival (months)^a^	181(173–189)	93(73–112)	<0.001
**Node-negative disease (n = 212)**	**n = 185 (90%)**	**n = 21 (10%)**	
Age (≤50/ >50 years)	58/132	10/12	0.157
Size (≤20/ 21-50/ >50 mm)	118/69/3	6/15/1	0.002
Grade (I / II / III)	36/66/88	1/9/12	0.180
ER status (no/yes)	50/66	22/16	0.114
PR status (no/yes)	58/58	23/15	0.261
HER2 status (no/yes)	158/32	16/6	0.228
Endocrine therapy (no/yes)	133/50	16/5	0.732
Chemotherapy (no/yes)	129/54	13/8	0.419
Tumour recurrence (no/local/distant/both)	163/9/15/3	12/0/10/0	<0.001
Alive/cancer death/non cancer death	125/24/41	3/11/8	0.001
Cancer specific survival (months)^a^	194(186–202)	110(75–146)	<0.001
**Triple-negative patients (n = 120)**	**n = 104(87%)**	**n = 16(13%)**	
Age (≤50/ >50 years)	44/60	6/10	0.718
Size (≤20/ 21-50/ >50 mm)	59/40/5	4/11/1	0.037
Grade (I / II / III)	1/16/87	0/5/11	0.212
Involved lymph node (-ve/+ve)	65/39	5/11	0.019
Endocrine therapy (no/yes)	92/12	15/0	0.167
Chemotherapy (no/yes)	45/59	6/9	0.812
Tumour recurrence (no/local/distant)	81/3/20	7/0/9	0.002
Alive/cancer death/non cancer death	64/24/16	2/13/1	0.014
Cancer specific survival (months)^a^	172(157–186)	64(37–92)	<0.001

^a^ = Mean (95% CI).

### Survival analysis of LBVI_H_&_E_, LVI_D2-40_ and BVI_FVIII_ in the whole cohort, in node-negative patients and in triple-negative patients

The minimum follow-up of survivors was 142 months; median follow-up of survivors was 168 months. During follow up 171 patients died, 97 died of their cancer. The presence of LBVI_H_&_E_, LVI_D2-40_ and BVI_FVIII_ were analysed with 15 years follow-up data using the Kaplan–Meier analysis and Cox regression.

**Figure 2:**
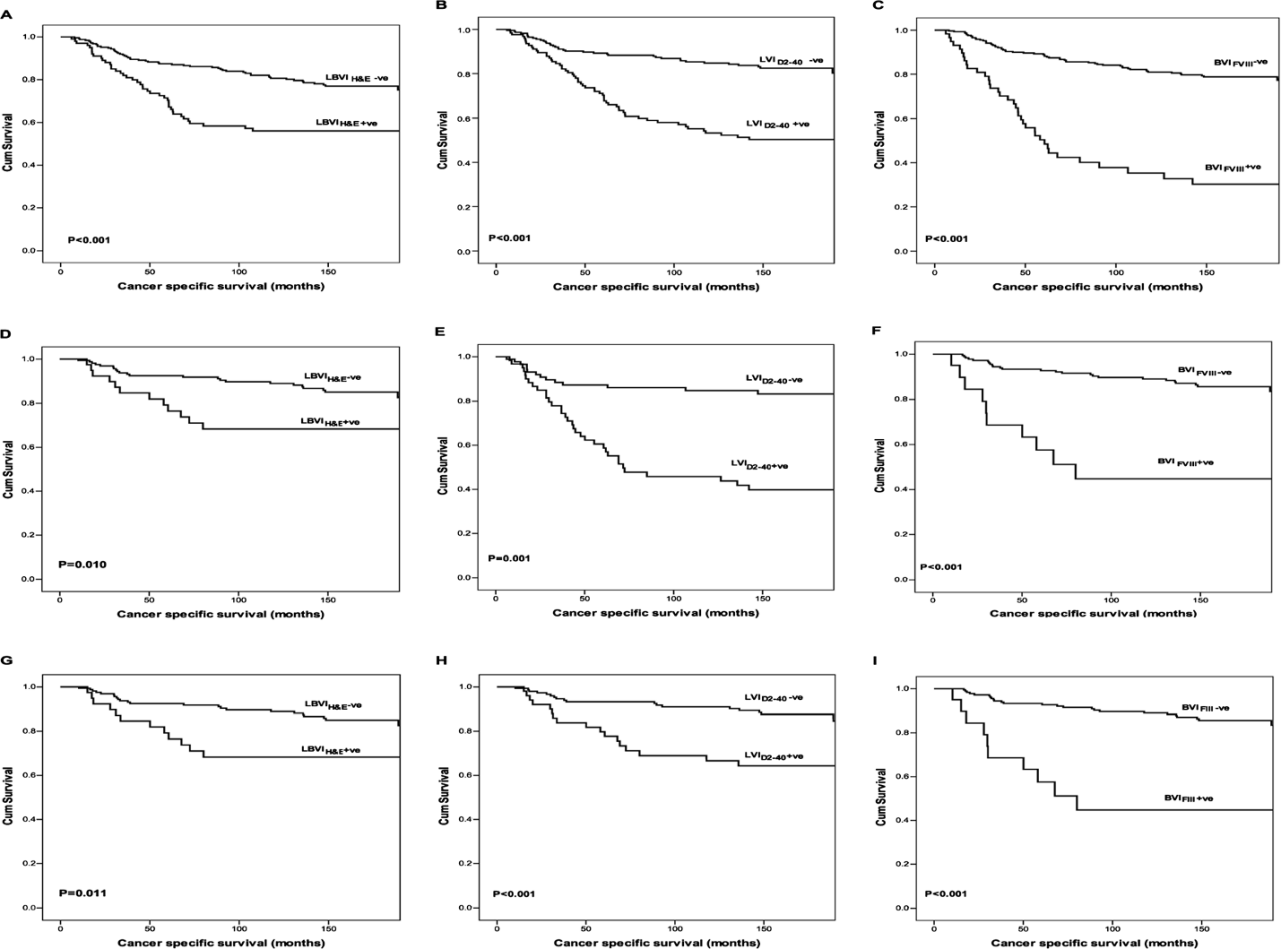
**Comparison of Kaplan-Meier survival curves (Log rank) of cancer specific survival for LBVI**_**H**_ **&**_**E**_ **, LVI**_**D2-40**_ **and BVI**_**FVIII**_ **in whole cohort (A-C), node-negative patients (D-F) and triple-negative patients (G-I).**

Kaplan–Meier curves showed increased risk of death with LBVI_H_&_E_, LVI_D2-40_ and BVI_FVIII_ in the whole cohort, node-negative and triple-negative patients (Figure 2). Univariate analysis indicated that LBVI_H_&_E_ was significantly associated with cancer specific survival in the whole cohort (*P* < 0.001), node-negative (*P* = 0.010) and in triple-negative patients (*P* = 0.011). The Presence of LVI_D2-40_ was strongly and significantly associated with cancer specific survival in the whole cohort (*P* < 0.001), in node-negative patients (*P* = 0.001) and in triple-negative patients (*P* < 0.001). The presence of BVI_FVIII_ was strongly and significantly associated with cancer specific survival in the whole cohort, node-negative and triple-negative patients (all P < 0.001) (Table 5).

**Table 5:** **The relationship between clinic-pathological characteristics and cancer specific survival in patients with primary operable invasive ductal breast cancer**

All patients (n = 360)	Univariate analysis		Multivariate analysis	
	Hazard ratio (95% CI)	***P***-value	Hazard ratio (95% CI)	***P***-value
Age (≤50/ >50 years)	0.97(0.64-1.45)	0.861		
Size (≤20/ 21-50/ >50 mm)	2.16(1.52-3.05)	<0.001	1.58(1.09-2.29)	0.014
Grade (I / II / III)	1.84(1.31-2.57)	<0.001	1.43(1.00-2.05)	0.049
Involved lymph node (-ve/+ve)	2.83(1.87-4.28)	<0.001	1.82(1.17-2.83)	0.008
ER (no/yes)	0.59(0.39-0.89)	0.012		0.571
PR (no/yes)	0.72(0.48-1.09)	0.128		
HER2 status (no/yes)	1.34(0.84-2.14)	0.216		
Endocrine therapy (no/yes)	0.48(0.26-0.88)	0.018		0.114
Chemotherapy (no/yes)	1.56(1.05-2.33)	0.029		0.611
LBVI_H_&_E_ (absent/present)	2.39(1.61-3.54)	<0.001		0.196
LVI_D2–40_ (absent/present)	3.31(2.19-4.97)	<0.001	1.69(1.08-2.67)	0.023
BVI_FVIII_ (absent/present)	5.12(3.38-7.78)	<0.001	3.35(2.21-5.63)	<0.001
**Node-negative patients (n = 212)**				
age (≤50/ >50 years)	0.69(0.36-1.36)	0.290		
Size (≤20/ 21-50/ >50 mm)	2.33(2.32-3.31)	0.007	1.93(1.04-3.59)	0.038
Grade (I / II / III)	1.64(1.64-2.74)	0.061		0.184
ER (no/yes)	0.75(0.38-1.45)	0.388		
PR (no/yes)	0.91(0.47-1.78)	0.780		
HER2 status (no/yes)	2.11(1.03-4.31)	0.040		0.368
Endocrine therapy (no/yes)	0.91(0.41-2.02)	0.822		
Chemotherapy (no/yes)	0.83(0.39-1.72)	0.612		
LBVI_H_&_E_ (absent/present)	2.43(1.21-4.89)	0.010		0.649
LVI_D2–40_ (absent/present)	3.24(1.67-6.29)	0.001	2.29(1.15-4.58)	0.004
BVI_FVIII_ (absent/present)	6.03(2.87-13.77)	<0.001	4.43(2.07-9.51)	0.001
**Triple-negative patients (n = 120)**				
age (<50/ >50 years)	1.09(0.57-2.01)	0.784		
Size (≤20/ 21-50/ >50 mm)	3.43(2.01-5.85)	<0.001	2.94(1.65-5.24)	<0.001
Grade (I / II / III)	0.79(0.39-1.58)	0.503		
Involved lymph node (-ve/+ve)	4.08(2.01-8.27)	<0.001	2.36(1.11-5.03)	0.026
Endocrine therapy (no/yes)	0.89(0.27-2.89)	0.842		
Chemotherapy (no/yes)	0.93(0.48-1.78)	0.824		
LBVI_H_&_E_ (absent/present)	2.31(1.21-4.42)	0.011		0.294
LVI_D2–40_ (absent/present)	3.57(1.82-7.04)	<0.001	2.61(1.36-5.04)	0.014
BVI_FVIII_ (absent/present)	4.68(3.09-10.31)	<0.001	3.63(1.38-6.56)	0.001

In multivariate survival analysis, tumour size (*P* = 0.014), LN status (*P* = 0.008), LVI_D2-40_ (*P* = 0.023) and BVI_FVIII_ (*P* < 0.001) remained independently associated with cancer specific survival. In multivariate survival analysis for node-negative patients, tumour size (*P* = 0.034), LVI_D2-40_ (*P* = 0.004) and BVI_FVIII_ (*P* = 0.001) remained independent predictors of shorter cancer specific survival. In multivariate survival analysis for triple-negative patients, tumour size (*P* < 0.001), LN status (*P* = 0.008), LVI_D2-40_ (*P* = 0.014) and BVI_FVIII_ (*P* = 0.001) remained independently associated with cancer specific survival (Table 5).

## Discussion

The results of the present study show that LBVI_H_&_E_, LVI_D2-40_ and BVI_FVIII_ all predicted tumour recurrence and cancer specific survival in an observational cohort of patients with early breast cancer. These results make a case for routine clinical assessment of lymphatic and blood vessel invasion by IHC to ascertain LVI and BVI.

In the present study, the proportion of patients with LBVI_H_&_E_ (28%) was consistent with most previous studies of breast cancer compared with (22-48%) in the literature, (20%) compared with (15-28%) for patients with node-negative tumour, and (29%) compared with (24-45%) for patients with triple-negative tumour [24]. Similarly, in terms of the association between LBVI_H_&_E_ and other well established high risk features such as tumour size, LN status, tumour grade, and breast cancer recurrence and survival are consistent with previous studies. Therefore, the present cohort is consistent with previous reports in which the prognostic value of LBVI_H_&_E_ has been established.

In the present study, the proportion of patients with LVI_D2-40_ (35%) was consistent with most previous studies using a similar approach (28-46%), (26%) compared with (15-28%) for patients with node-negative tumour, and (38%) compared with (26-41%) for patients with triple-negative tumour [24]. LVI_D2-40_ was associated with other well established high risk features such as tumour size, LN status, tumour grade, and with tumour recurrence. In addition, the presence of LVI_D2-40_ was significantly associated with reduced hormonal treatment and increased chemotherapy.

Furthermore, the presence of LVI_D2-40_ provided independent prognostic information not only in the whole cohort but also in the subgroup of patients with lymph node-negative and triple-negative breast cancer. These results are consistent with recent studies that assessed LVI objectively using D2-40 [16, 17, 27, 28]. Thus, the present study confirms that D2-40 staining is a practical and effective way of identifying endothelial cells lining lymphatic vessels in patients with early breast cancer, in particular node-negative disease. These findings suggest that LVI_D2-40_ might usefully be incorporated into the routine clinical pathological staging of patients with breast cancer.

In the present study, the proportion of patients with BVI (Factor VIII) was lower than that of previous studies by Kato and colleagues that used a similar approach (16%) compared to (27-29%) in the whole cohort and (10%) compared to (18%) in node-negative patients [15, 29, 30]. Given that Kato and colleagues did not use a specific lymphatic marker such as D2-40 to differentiate between lymphatic and blood vessels and that Factor VIII has been found to be occasionally reactive to lymphatic endothelium, it may be that the higher rate reported by Kato and co-workers reflects LVI being assessed as BVI. Moreover, the present cohort would not explain the large discrepancy between the present BVI rate and that reported by Mohammed and colleagues [16, 17] of only 0.7% of cases. Clearly, further prospective work is required across multiple centres to standardise the reporting of BVI, an important determinant of outcome in primary operable ductal breast cancer.

The results of the present study show for the first time the significance of BVI in triple-negative breast cancer. This is an important finding, because currently used clinic-opathologic and molecular markers, including the recent multigene assays, have a limited prognostic value in this molecular subtype. Most of these tumours are of high grade and exhibit poor prognosis gene signatures [31–33]. Thus objective assessment of BVI may provide additional independent prognostic information for this clinically important subgroup, in whom risk stratification and decisions about systemic therapy need to be determined.

The results of the present study suggest that BVI is less frequent than LVI in breast cancer, consistent with previous studies [15, 22, 34, 35]. This would suggest that LVI is potentially a more important route of breast cancer spread. However, results of the present study show that twenty one of 212 patients (10%) without lymph node metastases had BVI. Blood vessel invasion in patients without lymph node metastases may explain the subsequent development of metastatic disease.

It is recognised that D2-40 may stain myoepithelial cells of the normal breast ducts and ductal carcinoma *in situ* (DCIS) especially in small ducts completely filled by solid-pattern DCIS [36–38]. There is evidence that p63 staining may be useful in distinguishing D2-40 positive myoepithelium. However, this would increase the complexity of the present approach for routine clinical pathological analysis. Moreover, with awareness that myoepithelium may also be immunoreactive largely obviates this problem. Specifically, the tumour growth pattern enables distinction of ductal carcinoma *in situ* from lymphovascular invasion. Also, the myoepithelium is discontinuous in small ducts whereas the endothelial lining of the lymph vessels is continuous and the myoepithelial cells of larger ducts are larger than the endothelial cells of lymph vessels [39]. Finally, the distribution of the stain for the myoepithelial cells is recognised to be patchy and the intensity less than that of the adjacent lymphatic endothelium [40]. Therefore, increase in sensitivity of detection of lymph vessel invasion may be reasonably attributed to the demarcation of lymphatic endothelium that stains positively for D2-40 around the tumour emboli and although, D2-40 may also bind to myoepithelium of breast ducts, it is not difficult to distinguish between myoepithelial reactivity and endothelial staining of the vessels.

Factor VIII has been previously reported as a blood vessel endothelial marker in breast cancer and is consistently found in normal endothelial cells in blood vessels. While it occasionally stains endothelial cells in lymphatics, staining of lymphatic endothelium is usually faint and discontinuous [21, 29, 41]. Some studies have suggested that the vascular marker CD31 may be superior to factor VIII for blood vessels staining [42, 43]. However, another study reported that the higher sensitivity of CD31 of vascular endothelium did not yield results more discriminating for predicting survival outcome than results produced with factor VIII [44].

In the present study, although the value of lymphovascular invasion detected using IHC was significantly correlated with the value of lymphovascular invasion detected using H&E (*P* < 0.001), 80 (22%) patients that were negative for lymphovascular invasion on H&E showed positive LVI_D2–40_ and/or BVI_FVIII_, indicating that the frequency of detection of lymphovascular invasion increased using IHC. These lesions were difficult to identify on the H&E sections due to invasion into small lymphatic or blood vessels or due to vessels that had been obscured by tumour cells. Thirty four patients had tumours that were LBVI_H_&_E_ positive, were negative for both LVI_D2–40_ and BVI_FVIII_. A recognised explanation for such a discrepancy is that stromal retraction artifacts, caused by tissue handling and fixation, on H&E sections cause false positives [7, 21–23]. In addition, the H&E approach has considerable interobserver variability and lower overall detection rate in most previous studies [24]. The significance of this is that although AJCC guidelines mandate the reporting of lymphatic and blood vessel invasion, they lack a routine standardized pathological methodology to reliably report them.

The results of the present and previous studies [24] point to a substantial improvement in the consistency of reporting and an increase in the rate of detection of LBVI, LVI and BVI in patients with breast cancer cases using an IHC approach. Such an improvement has been documented with lymphatic (eg, podoplanin/D2-40) and blood vessel (eg, CD34 and CD31) endothelial markers. Moreover, these markers not only discriminate retraction artifacts from LVI and BVI but also distinguish between lymph vessels and blood vessels, allowing specifically study of LVI and BVI [7, 16, 21, 36, 39].

A limitation of the present study was that intra- and peritumoral LBVI foci were not separately analysed owing to small number of cases with intratumoural LBVI_H_&_E_ (5%) compared to the (95%) of peritumoural LBVI_H_&_E_. This precluded meaningful analysis of each component but was unlikely to materially influence the concordance between the detection of LBVI-H&E and LBVI-IHC. Although, several previous studies have reported the prognostic significance of LBVI using H&E staining these studies have not discriminated between the types of vessel invasion whether lymphatic or blood vessel and have inconsistently used the terms vascular or lymphovascular invasion. For example, the American Joint Committee on Cancer (AJCC) staging guidelines (2005) has used the term lymphovascular invasion to indicate both lymphatic and vascular involvement [20]. This clearly may be confusing as these terms may indicate involvement of lymphatic or lymphatic and blood vessels. This is largely a pragmatic approach to the limitations of the routine use of H&E slides to assess lymphovascular invasion. Another limitation was that the well established factors such as grade and ER status were not independently associated with cancer specific survival in all patients and in those with node-negative or triple-negative disease. This may suggest that the sample size was rather small for such multivariate analysis. Nevertheless, the results are of interest and make a case for further studies of routine clinical assessment of lymphatic and blood vessel invasion by IHC to ascertain LVI and BVI.

## Conclusions

In summary, the results of the present study show that IHC for D2-40 and Factor VIII define lymphatic and blood vessel invasion with greater sensitivity and specificity than H&E, improving detection of LVI and BVI in early invasive breast cancer. Moreover, the prognostic significance of the LVI_D2–40_ and BVI_FVIII_ was superior to that of LBVI_H_&_E_ and this was consistent throughout analysis of sub-cohorts. Therefore, these results make the case for their assessment in routine clinic-pathological practice.
